# Astaxanthin-Mediated Bacterial Lethality: Evidence from Oxidative Stress Contribution and Molecular Dynamics Simulation

**DOI:** 10.1155/2021/7159652

**Published:** 2021-12-09

**Authors:** Jamiu Olaseni Aribisala, Sonto Nkosi, Kehinde Idowu, Ismaila Olanrewaju Nurain, Gaositwe Melvin Makolomakwa, Francis O. Shode, Saheed Sabiu

**Affiliations:** ^1^Department of Biotechnology and Food Science, Faculty of Applied Sciences, Durban University of Technology, Durban, South Africa; ^2^Department of Pharmacology, The University of Minnesota Medical School, Minneapolis, USA

## Abstract

The involvement of cellular oxidative stress in antibacterial therapy has remained a topical issue over the years. In this study, the contribution of oxidative stress to astaxanthin-mediated bacterial lethality was evaluated *in silico* and *in vitro*. For the *in vitro* analysis, the minimum inhibitory concentration (MIC) of astaxanthin was lower than that of novobiocin against *Staphylococcus aureus* but generally higher than those of the reference antibiotics against other test organisms. The level of superoxide anion of the tested organisms increased significantly following treatment with astaxanthin when compared with DMSO-treated cells. This increase compared favorably with those observed with the reference antibiotics and was consistent with a decrease in the concentration of glutathione (GSH) and corresponding significant increase in ADP/ATP ratio. These observations are suggestive of probable involvement of oxidative stress in antibacterial capability of astaxanthin and in agreement with the results of the *in silico* evaluations, where the free energy scores of astaxanthins' complexes with topoisomerase IV ParC and ParE were higher than those of the reference antibiotics. These observations were consistent with the structural stability and compactness of the complexes as astaxanthin was observed to be more stable against topoisomerase IV ParC and ParE than DNA Gyrase A and B. Put together, findings from this study underscored the nature and mechanism of antibacterial action of astaxanthin that could suggest practical approaches in enhancing our current knowledge of antibacterial arsenal and aid in the novel development of alternative natural topo2A inhibitor.

## 1. Introduction

Antibiotic resistance in bacteria has become a significant public health threat, resulting in high mortality and morbidity each year [1]. Due to this resistance, bacterial infections have remained difficult to treat, and even the viable options such as combination therapy have posed increased risk of adverse events in patients [1, 2]. Faced with this threat, immediate action is required to develop novel antibacterial agents that could act via new mechanisms against infections caused by multidrug-resistant microorganisms. Recently, the involvement of cellular oxidative stress in antimicrobial-mediated antibacterial therapy has been opined as one of the novel alternatives of antibacterial actions [3, 4]. In this context, the antibacterial agents generate diverse forms of reactive oxygen species (ROS) while interacting with their targets [5], and the fluoroquinolones are one of the implicated classes of antibacterials utilizing this mechanism. The fluoroquinolones (e.g., ciprofloxacin and novobiocin) target the topoisomerase 2As (topo 2As: DNA gyrase and topoisomerase IV) required for nucleic acid synthesis and transcription in bacteria [6, 7], and their multiple discrete binding sites such as the ATP-binding subunits on DNA gyrase have been recognised as important targets of synthetic and natural inhibitors [8]. While interacting with topo2As of bacteria, the fluoroquinolones boost electron transport chain activity, and this results in elevated production of ROS, which contributes to either cellular damage or death [4]. Despite this remarkable mechanism of antibacterial action of the fluoroquinolones, their applications have been limited in clinical practice due to the continuous occurrence of resistant microbes and associated adverse effects [9]. While efforts have been made to modify chemical moieties as improved versions of fluoroquinolones, no synthetic or natural inhibitors of topo2As have reached the clinic to date. Hence, the ATP-binding subunits of topo2As under altered cellular redox state represent attractive targets that could be unexploited to develop novel antibacterials that would help in combating the ever-increasing levels of multidrug-resistant-bacterial infections.

**Figure 1:**
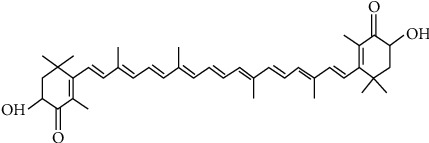
Structural representation of astaxanthin.

The level of ROS contribution to the bactericidal activity of antibacterials has been demonstrated to depend on the nature of compound [10]. In some compounds such as quinolone, rapid killing has been demonstrated to be fully through ROS generation while in some other compounds; other mechanisms are implicated [10, 11]. Interestingly, this concept of bacterial killing through ROS generation has also been implicated in some plant-derived phytochemicals such as phenolic acids and flavonoids [3, 4, 12]. Through autoxidation, these compounds generate high amount of ROS when catalyzed by transition metals. Although, the ROS generated in this manner such as superoxide ion and H_2_O_2_ are not too reactive and as such do not cause oxidative damage to bacterial macromolecules [13, 14]. However, through Fenton reaction (Fe^2+^ + H_2_O_2_ → Fe^3+^ + ·OH + OH), these ROS react with independent ferrous ion in bacterial cells to form OH^∗^ which are very reactive and can cause damage to bacterial macromolecules such as protein, lipid, and DNA and as such contribute to the ultimate death of the organism [13, 14]. Hence, exploring plant-derived compounds for their bactericidal capability through ROS generation remains a plausible area of research for identification of antibacterial agents whose mechanisms of action will rely on ROS generation. Consistent with the phenolics, the antibacterial activities of carotenoids have also been reported [13, 15] and more specifically, existing data on astaxanthin (Figure 1), a xanthophyll carotenoid, showing promising antibacterial activity against clinical isolates, wild- and mutant-typed cultures have been reported [13, 16]. However, no studies have linked ROS involvement in the bactericidal activity of astaxanthin or its inhibitory effect on topo2As (druggable targets) which have been demonstrated to facilitate ROS generation when interacting with the fluoroquinolones. Hence, for the first time, this study employed computational techniques in investigating the susceptibility of each of the topo2As subunit (DNA gyrase A&B and topoisomerase IV ParC&E) to astaxanthin while establishing the extent of ROS involvement in astaxanthin-mediated bacterial lethality *in vitro*.

## 2. Methodology

### 2.1. Computational Analyses

#### 2.1.1. Ligand and Protein Preparation

The 3D structures of the reference antibiotics (ciprofloxacin (CID: 2764), novobiocin (CID: 54675769)) and astaxanthin (CID: 5281224) were obtained from PubChem (https://www.pubchem.ncbi.nlm.nih.gov) and then optimized by adding Gasteiger charges and nonpolar hydrogen atoms using the Avogrados software in preparation for docking [17]. Similarly, the crystal structures of DNA gyrase (Gyr) subunits A (ID: 4CKK) and B (ID: 4DUH) and topoisomerase IV (ParC (ID: 1ZVU) and ParE (ID: 1S14)) were obtained from the Protein Data Bank (https://www.rcsb.org/pdb/), followed by structural optimization involving removal of water molecules, nonstandard amino acids, and heme using UCSF Chimera v1.14 [17]. The structures generated were then saved in a PDB format and used for molecular docking.

#### 2.1.2. Grid Preparation, Molecular Docking, Dynamics, and Postdynamics Simulation

Prior to docking, the binding sites of the DNA GyrA and GyrB as well as topoi IV ParC and ParE were determined as earlier reported [18], and the grid boxes covering the binding sites in each case were generated with well-defined *x*-*y*-*z* coordinates (Table S1). The optimized 3D structures of ligands (ciprofloxacin, novobiocin, and astaxanthin) and proteins were thereafter docked using the Autodock vina 1.1.2 software in Chimera v1.14 [18]. The molecular docking was evaluated according to the free binding energy of the ligands with the respective proteins, prior to pose ranking for fitness within the binding pocket of each protein for continuum and discrete bond interactions [17]. Thereafter, molecular dynamics simulation (MDS) was performed on the ligand-protein complexes with the best docking scores in each case [19].

The post-dynamic data was examined as previously described [20], and analysis of radius of gyration (ROG) and root mean square deviation (RMSD) were done followed by evaluation of the free binding energy and comparing the binding affinity of the resulting complex in each scenario of the simulation. The MDS was averaged among 100000 snapshots taken from a 60 ns MDS, and using the expression Δ*G*_bind_ = *G*_complex_–(*G*_Receptor_ + *G*_ligand_), the free binding energy (Δ*G*) was estimated. The ligand-receptor complexes' interaction at the active sites in each treatment case was identified post-MDS and visualized using Discovery Studio version 21.1.0.

### 2.2. Pharmacokinetic Properties

The SwissADME web tool (http://swissadme.ch/index.php) and Molinspiration online toolkit (https://www.molinspiration.com/cgi-bin/properties) were utilized to make predictions for the physicochemical and drug-likeness properties of astaxanthin while the Protox II webserver (https://tox-new.charite.de/protox_II/) which contains models for predicting toxicological endpoints related with a chemical structure was employed to determine its toxicity profiles.

### 2.3. *In Vitro* Evaluation

#### 2.3.1. Strains and Culture Conditions

The stocks of Gram-positive (*S. aureus*, *B. cereus*) and Gram-negative (*P. aeruginosa*, *E. coli*) strains used in this study were obtained from Microbiologics (Minnesota, USA) and subsequently propagated on Mueller-Hinton broth (MH) for 24 h at 37°C before use.

#### 2.3.2. Antibacterial Assays

The minimum inhibitory concentrations (MIC) of astaxanthin and the reference standards (ciprofloxacin and novobiocin) were evaluated as previously reported [21]. A range of 64 *μ*g/ml to 0.125 *μ*g/ml were prepared from the stock solutions of 128 *μ*g/ml of astaxanthin and the antibiotics. Subsequently, the prepared concentrations in each case were suspended in inocula (10^−4^ CFU/ml) in microtitre plates (96 wells), before incubation (37°C, 24 h). Judging by the absence of turbidity, the MIC in each case was taken as the lowest concentration of astaxanthin and reference antibiotics which inhibit bacterial growth.

For the bactericidal concentration (MBC), the method of Oloyede et al. [22] was employed. In brief, from the MIC plate with no visible growth, 100 *μ*l bacterial suspensions were taken and subcultured on nutrient agar. The plates were incubated (37°C, 48 h), and the lowest concentration of astaxanthin and reference antibiotics that showed no observable growth was selected as the MBC.

#### 2.3.3. Time-Kill Susceptibility Assay

The time-dependent susceptibility of test organisms to astaxanthin was investigated as previously described [23]. Briefly, the test organisms were grown overnight in MH broth, centrifuged, and resuspended in 50 ml fresh MH medium (Optical Density_600_ = 0.1) after which it was grown aerobically at 37°C to optical density of 0.2. The cultures were then incubated with astaxanthin, ciprofloxacin, and novobiocin at a concentration of 4x MIC, at 37°C for 3 h, with dimethyl sulfoxide (DMSO) serving as a control. Following that, the absorbance in each case was measured at 600 nm every 30 min for a total incubation time of 3 h.

#### 2.3.4. ROS Monitoring Assays

*(1) Superoxide Anion Assay*. The method of Ajiboye et al. [24] was employed for the superoxide anion generation assay. In brief, 1 ml of the exponential phase of test organisms was incubated for 30 min with 4x MIC of either astaxanthin, ciprofloxacin, or novobiocin, before nitroblue tetrazolium (0.5 ml, 1 mg/ml) addition and incubation (37°C, 30 min). Subsequently, the mixture was centrifuged (1,500 × *g*, 10 min) after HCl (0.1 ml) addition. With DMSO and phosphate-buffered saline (0.8 ml, pH 7.5), the reduced nitroblue tetrazolium in the pellets was extracted and diluted, and the absorbance (575 nm) was taken, while the molar extinction coefficient of MTT (5-diphebyl tetrazolium bromide) used to estimate the cells' superoxide anion level.

*(2) Hydroxyl Radical Assay*. This was done using the method of Oloyede et al. [22]. Using MH broth medium, overnight grown bacterial cultures were harvested and resuspended in MH medium (50 ml, optical density_600_ = 0.1) and aerobically grown to optical density_600_ of 0.2 at 37°C before addition of 2,2′-dipyridyl (500 *μ*mol/L) and/or 4x MIC astaxanthin, ciprofloxacin, or novobiocin followed by incubation (37°C, 3 h). At every 30 min incubation time, an absorbance reading at 600 nm was taken.

#### 2.3.5. Oxidative Stress Biomarker Assays

*(1) Glutathione Assay*. Cells were tested for reduced glutathione (GSH) using the instructions in GSH assay kit. The cells were treated for 30 min at 37°C with 4x MICs of either ciprofloxacin, novobiocin, or astaxanthin. After treatment, they were pelleted, rinsed, frozen, and thawed twice and later centrifuged (10,000 × *g*, 10 min). The working reagent was mixed with 10 *μ*l of cell free extract, and the blank was made with 5% sulfosalicylic instead of bacterial cells. The absorbance (412 nm) was taken using a microplate reader, and the GSH standard curve was used to determine the GSH concentration in the cell free extract [25].

*(2) ADP/ATP Assay*. In this case, the ADP/ATP test kit was used as per manufacturer's instructions. The 4x MIC of either ciprofloxacin, novobiocin, or astaxanthin was incubated (30 min, 37°C) with cells in exponential phase before the cells (90 ml) were mixed with the ATP reagent and further incubated (1 min, 25°C). Thereafter, luminescence (relative light units (RLU_*a*_)) was measured for ATP and the mixture incubated further (10 min, 25°C) before luminescence reading (RLU_*b*_) [26]. Finally, 5 *μ*l of the ADP reagent was added vortexed, and a new luminescence (RLU_*c*_) was read 1 min later prior to the estimation of ADP/ATP ratio from the expression: ADP/ATP= (*RLUc* − *RLUb*)/*RLUa*.

### 2.4. Data Analyses

Experiments were performed in triplicates for the *in vitro* assessments, and the findings presented as the mean ± standard deviation of replicate experiments. Using the Analysis ToolPak in Microsoft Excel, the analysis of variance and the students *t*-test were done to identify significant difference at *p* < 0.05 level between treatment means. The plots for the computational analyses were constructed with Origin V18.

## 3. Results

### 3.1. Computational Analyses

The binding energy scores of the ligand-protein complexes generated through molecular docking are presented in Table 1, with the docked DNA GyrA- and topoi iv ParC-ciprofloxacin complexes having scores of -7.4 and -6.9 kcal/mol, respectively, while -8.8 and -8.4 kcal/mol were obtained with astaxanthin, respectively. On the other hand, the scores for DNA GyrB- and topoi iv ParE-novobiocin docked complexes were -8.7 and -6.6 kcal/mol, respectively, which were similar to -8.7 and -6.7 kcal/mol with astaxanthin, respectively (Table 1).

**Figure 2:**
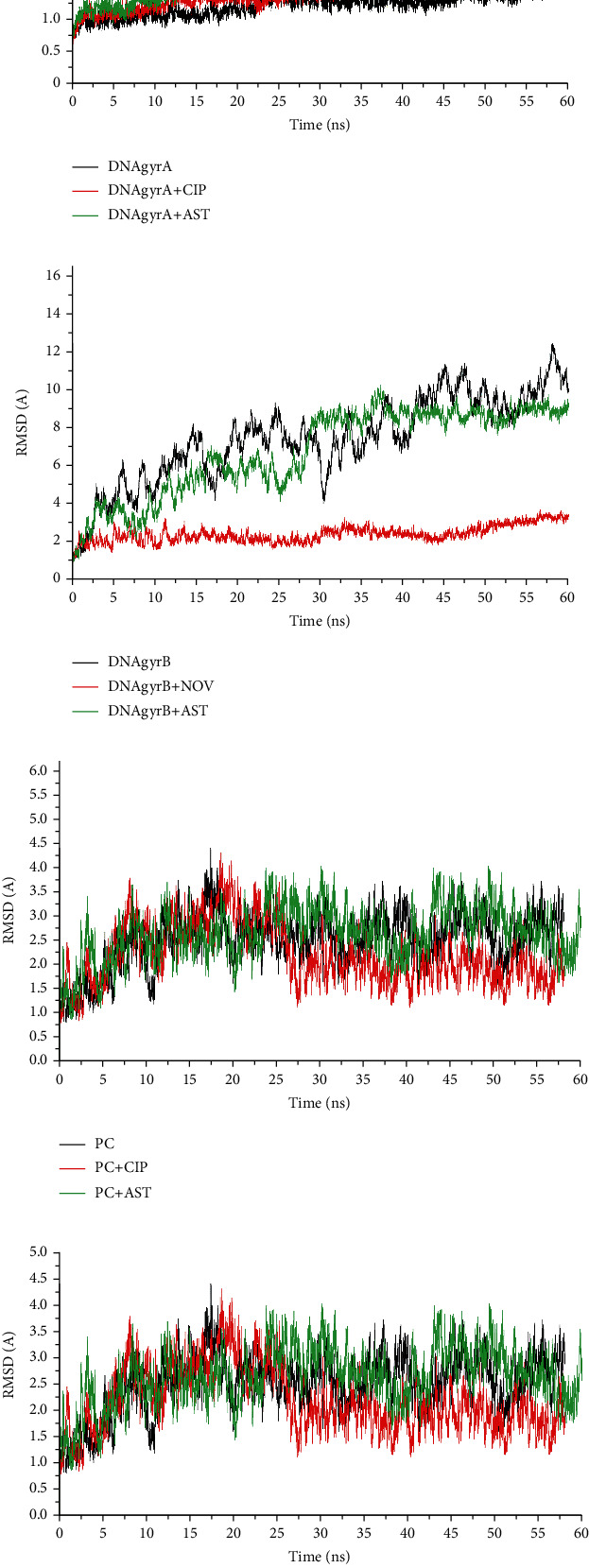
Comparative plots of alpha-carbon of (a) DNA gyrase A, (b) DNA gyrase B, (c) ParC, and (d) Par E, and astaxanthin and standard antibiotics (ciprofloxacin and novobiocin) presented as root mean square deviation (RMSD) over a 60 ns molecular dynamics simulation.

**Figure 3:**
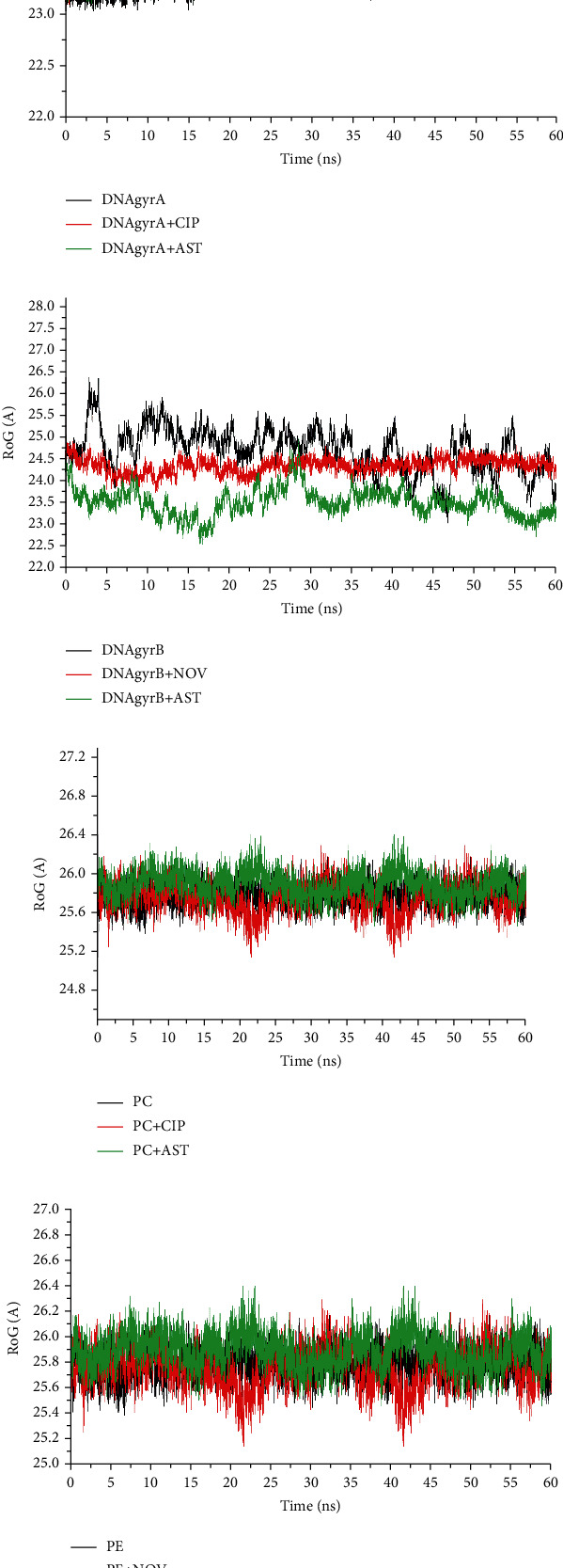
Comparative plots of alpha-carbon of (a) DNA gyrase A, (b) DNA gyrase B, (c) ParC, and (d) Par E, and astaxanthin and standard antibiotics (ciprofloxacin and novobiocin) presented as radius of gyration (RoG) over a 60 ns molecular dynamics simulation.

Table 2 shows the results obtained in terms of the estimated free binding energy of the complexes following MDS analysis. The negative binding free energy scores of astaxanthin-topoi iv ParC and topoi iv ParE complexes were -35.55 and -38.73 kcal/mol, respectively, which were higher than the reference antibiotics (Table 2). However, in complex with GyrA and GyrB, ciprofloxacin (-33.72 kcal/mol) and novobiocin (-56.52 kcal/mol) had higher negative binding free energy scores than astaxanthin (-28.840 and -24.42 kcal/mol, respectively). The average RMSD values of astaxanthin complexes with GyrA, PaC, and ParE were 1.40 Å, 2.31 Å, and 2.29 Å, respectively, which compared well with those of the reference antibiotics (1.21 Å, 2.05 Å, and 1.99 Å) and unbound protein (1.08 Å, 2.56 Å, and 2.45 Å) (Figures 2(a), 2(c), and 2(d)). However, in complex with DNA GyrB, the RMSD value for astaxanthin (4.03 Å) was lower than that of the unbound protein (5.00 Å) (Figure 2(b)). The data obtained with respect to RoG are presented in Figure 3. The average RoG values of astaxanthin complex with GyrA, GyrB, PaC, and ParE were 23.28 Å, 23.45 Å, 26.10 Å, and 26.09 Å, respectively, which were similar to those obtained with the reference antibiotics (23.52 Å, 24.35 Å, 25.47 Å, and 25.38 Å, respectively) and the unbound protein (23.20 Å, 25.45 Å, 25.95 Å, and 25.73 Å, respectively) (Figures 3(a)–3(d)).

**Figure 4:**
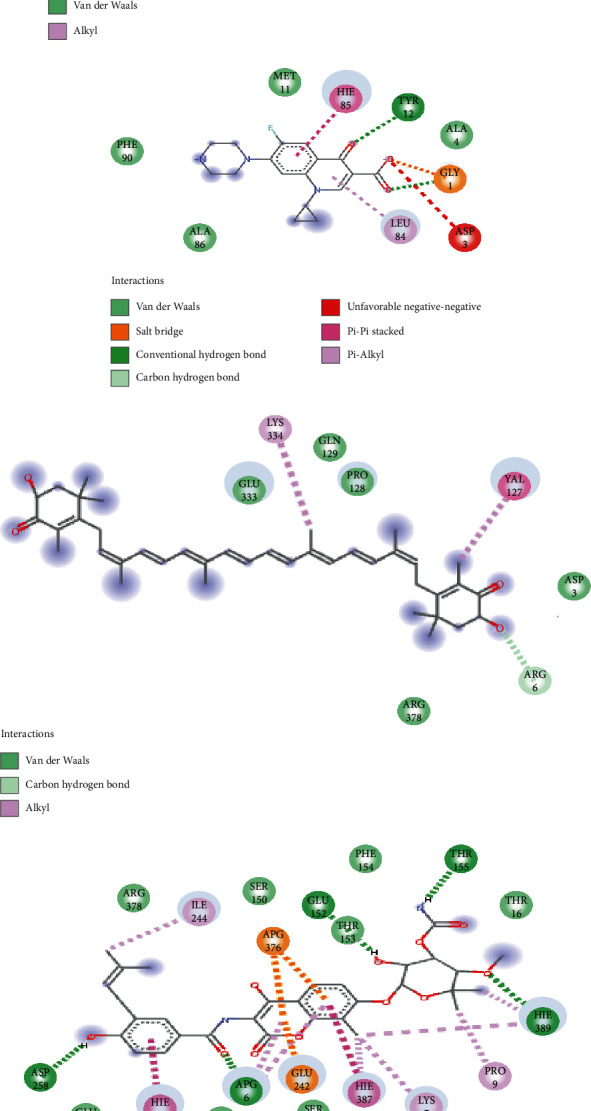
Interaction plots of (a) DNA gyrase A + astaxanthin, (b) DNA gyrase A + ciprofloxacin, (c) DNA gyrase B + astaxanthin, and (d) DNA gyrase B + novobiocin, post-60 ns of molecular dynamics simulation.

**Figure 5:**
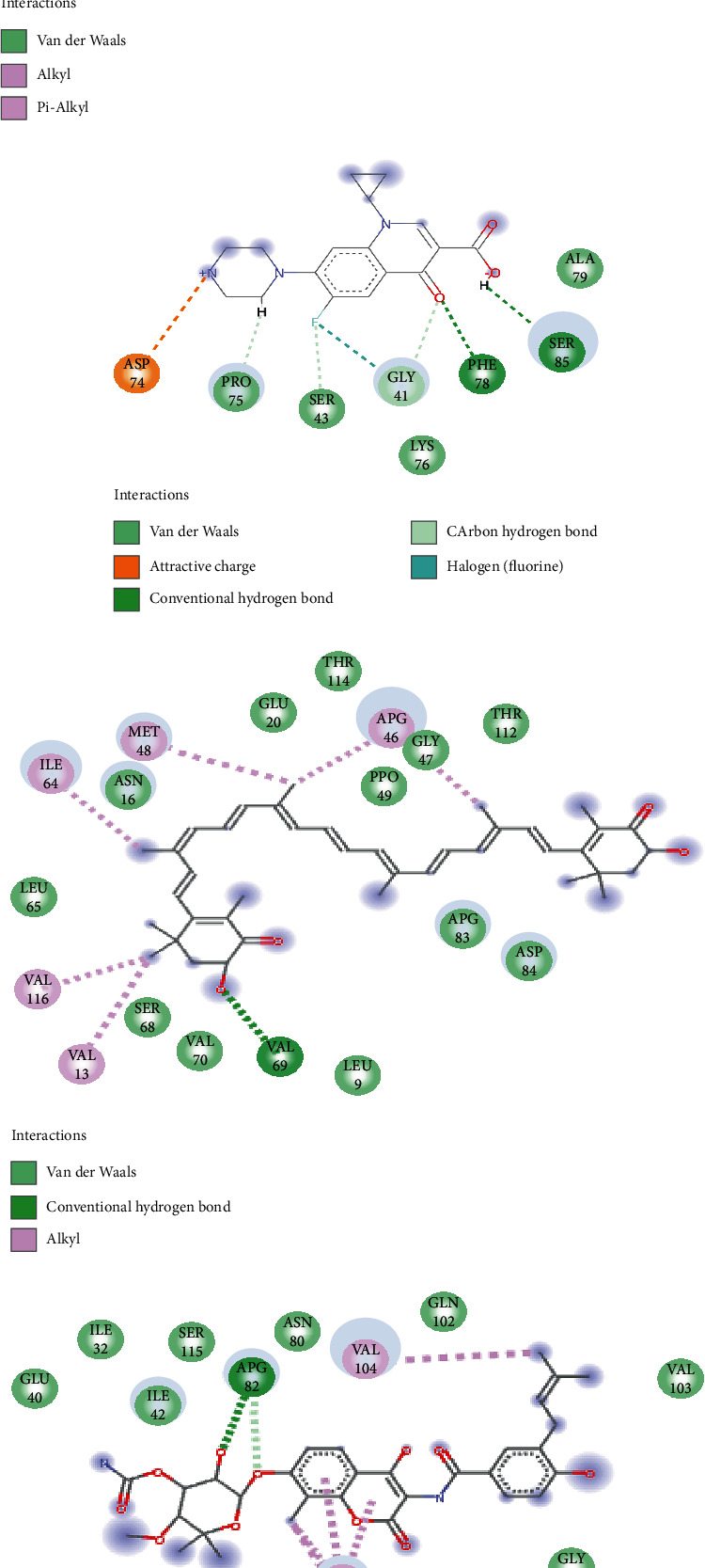
Interaction plots of (a) Topoi IV ParC + astaxanthin, (b) Topoi IV ParC + ciprofloxacin, (c) Topoi IV ParE + astaxanthin, and (d) Topoi IV ParE + novobiocin, post-60 ns of molecular dynamics simulation.

The interactions between the residues at the active site of the proteins with astaxanthin post-MDS revealed that ciprofloxacin (10) and novobiocin (26) had more interactions with GyrA and GyrB than astaxanthin with 7 and 8 interactions with GyrA and GyrB, respectively (Figure 4). Astaxanthin formed van der Waals interactions with Arg62, Ala39, Gly178, Lys43, Ala37, and Gly40 and alkyl bonds with (Leu38) of DNA GyrA while forming van der Waals interactions with Asp3, Arg378, Arg6, Pro128, Gln129, and Gln333; alkyl bonds with Val127 and Lys334 and hydrogen bond with Arg6 of DNA GyrB (Figure 4). However, astaxanthin with 17 and 19 interactions with Topoi IV ParC and Topoi IV ParE, respectively, were higher than that of ciprofloxacin (9) and novobiocin (16) against Topoi IV ParC and Topoi IV ParE, respectively (Figure 5). Astaxanthin formed van der Waals interactions with Tyr46, Ala79, Asp32, Arg28, Val33, Gly35, Phe78, Hie 38, Pro39, Leu34, Asp42, Gly41, and Ser43 and alkyl bonds with Lys36, Cys45 Hie40, and Pro75 of Topoi IV ParC while forming van der Waals interactions with Leu9, Asp84, Arg83, Val70, Ser68, Leu65, Pro49, Thr112, Thr114, Glu20, and Asn16; alkyl bonds with Val13, Val116, Ile64, Met48, Arg46(2); and hydrogen bond with Val69 of Topoi IV ParE (Figure 5). Astaxanthin had no common amino acid residues with ciprofloxacin and novobiocin with DNA GyrA and Topoi IV ParE, respectively, but had common interactions with ciprofloxacin and novobiocin with amino acid residues Pro75, Ser43, Gly41, Phe78 of Topoi IV ParC and Arg378, and Arg6 of DNA GyrB, respectively.

The ADMET (absorption, distribution, metabolism, excretion, and toxicity) properties of astaxanthin and reference antibiotics are shown on Table 3. Astaxanthin has a molecular weight, hydrogen bond donor, and hydrogen bond acceptor of 596.84 g/mol, 2, and 4, respectively, which were lesser than that of novobiocin (612.62 g/mol, 11, and 5, respectively). For bioavailability and solubility in water, astaxanthin compared well with novobiocin with a score of 0.17 and moderate solubility, respectively, relative to 0.55 with high solubility for ciprofloxacin. Astaxanthin was a noninhibitor of all the CYP isoenzymes; however, novobiocin inhibit the CYP3A4. Also, astaxanthin passed the common toxicity tests while novobiocin and ciprofloxacin were predicted to be potential immunotoxin and mutagen, respectively. Judging by the estimated LD_50_ values, astaxanthin (4600 mg/kg) was a class 5 drug contrary to the antibiotics that belong to class 4 (Table 3).

### 3.2. *In Vitro* Evaluations

**Figure 6:**
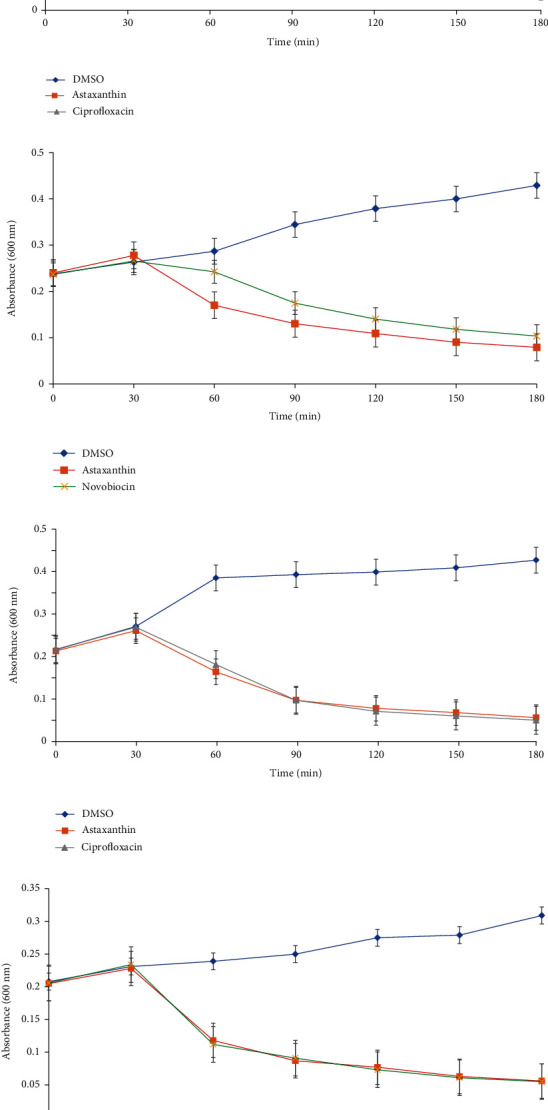
Viability of (a) *E. coli*, (b) *P. aeruginosa*, (c) *B. cereus*, and (d) *S. aureus* exposed to astaxanthin (4x MIC).

The results of the antimicrobial activity of astaxanthin against the test organisms are shown in Table 4. The MIC value for astaxanthin against *B. cereus*, *E. coli*, and *P. aeruginosa* was 16 *μ*g/ml relative to a range of 0.25–0.125 *μ*g/ml for the reference standards. However, the MIC of astaxanthin against *S. aureus* was lower than that of novobiocin. Furthermore, astaxanthin had the same MBC value against *B. cereus*, *E. coli*, and *P. aeruginosa* as novobiocin, which was lower than the values observed with ciprofloxacin against all the tested organisms (Table 4). Regarding the time-kinetics, all the astaxanthin-treated bacterial strains demonstrated a concentration-dependent effect observed as decrease in absorbance after 30 min of incubation, compared to the DMSO-treated strains (Figure 6). However, while the decrease in absorbance in astaxanthin-treated *P. aeruginosa* was more pronounced than the observation with novobiocin (Figure 6(b)), there was no significant difference in the effect elicited by astaxanthin against *S. aureus* and *B. cereus* when compared to the reference antibiotics (Figures 6(c) and 6(d)).

**Figure 7:**
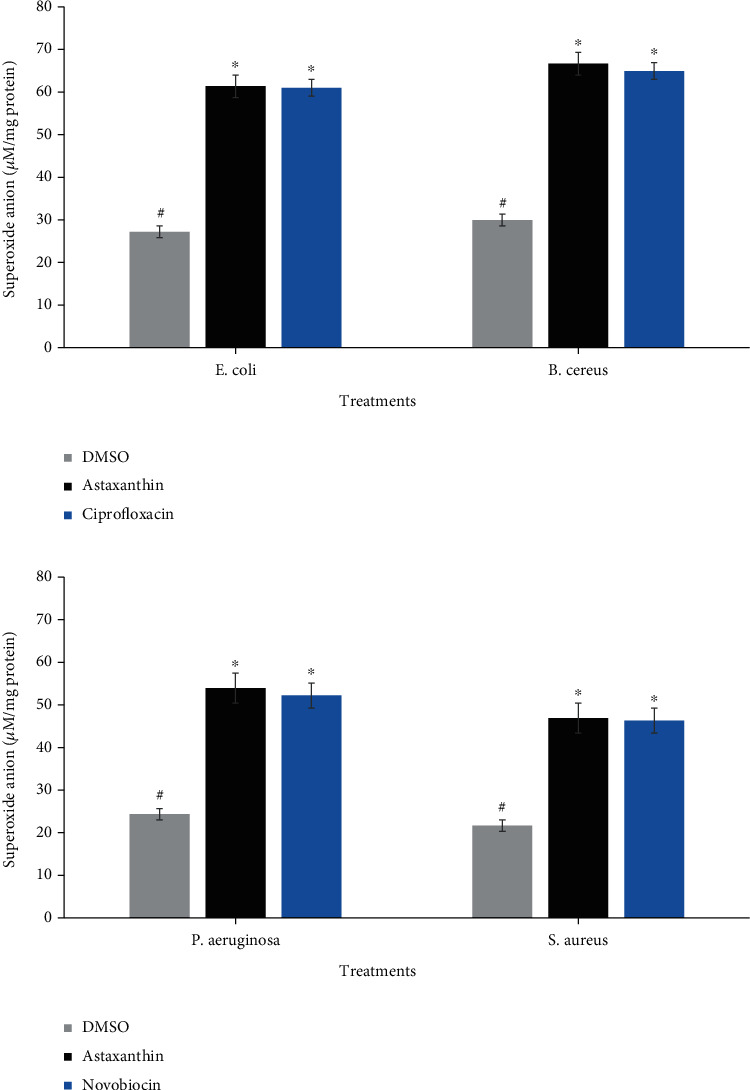
Generated superoxide anion radicals in astaxanthin- (a) and ciprofloxacin-treated *E. coli* and *B. cereus* cells (b) and novobiocin-treated *P. aeruginosa* and *S. aureus* cells. ^#^^∗^Bars carrying the same symbol are not significantly different (*p* > 0.05).

**Figure 8:**
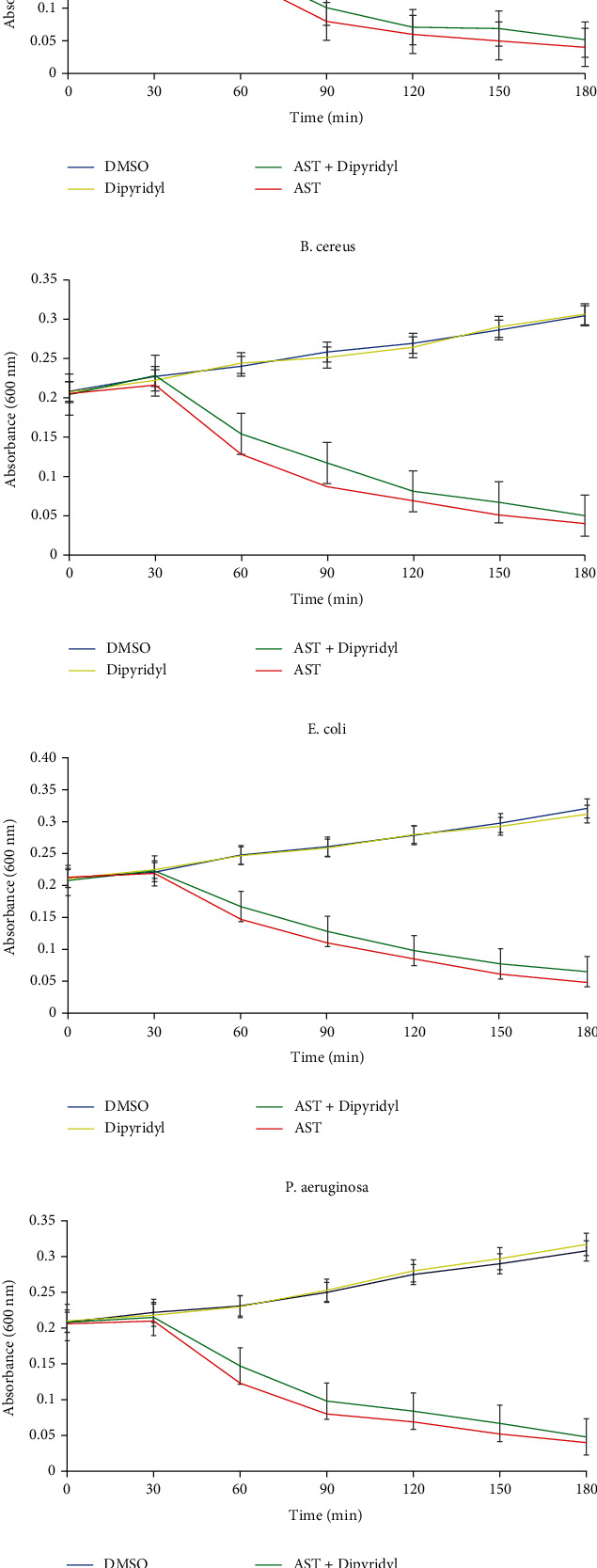
Involvement of hydroxyl radical in astaxanthin (4x MIC) mediated lethality on (a) *B. cereus*, (b) *S. aureus*, (c) *E. coli*, and (d) *P. aeruginosa*, in the presence of 2,2′-dipyridyl (500 *μ*mol/L).

The superoxide anion radicals generated during astaxanthin treatment of the bacterial cells are shown in Figure 7. The superoxide anion levels of *P. aeruginosa*, *B. cereus*, *S. aureus*, and *E. coli* increased significantly following treatment with astaxanthin, when compared with DMSO-treated cells. It was noteworthy that the levels of superoxide anion generated in astaxanthin-treated cells compared favorably with those generated in cells treated with ciprofloxacin (Figure 7(a)) and novobiocin (Figure 7(b)). The time-killing rate as a result of treatment of bacterial cells with astaxanthin in the presence and absence of 2,2′dipyridyl is shown in Figure 8. Cotreatment of bacterial cells with astaxanthin and 2,2′dipyridyl resulted in marginally decreased time-dependent killing of the bacterial cells relative to treatment with astaxanthin only.

**Figure 9:**
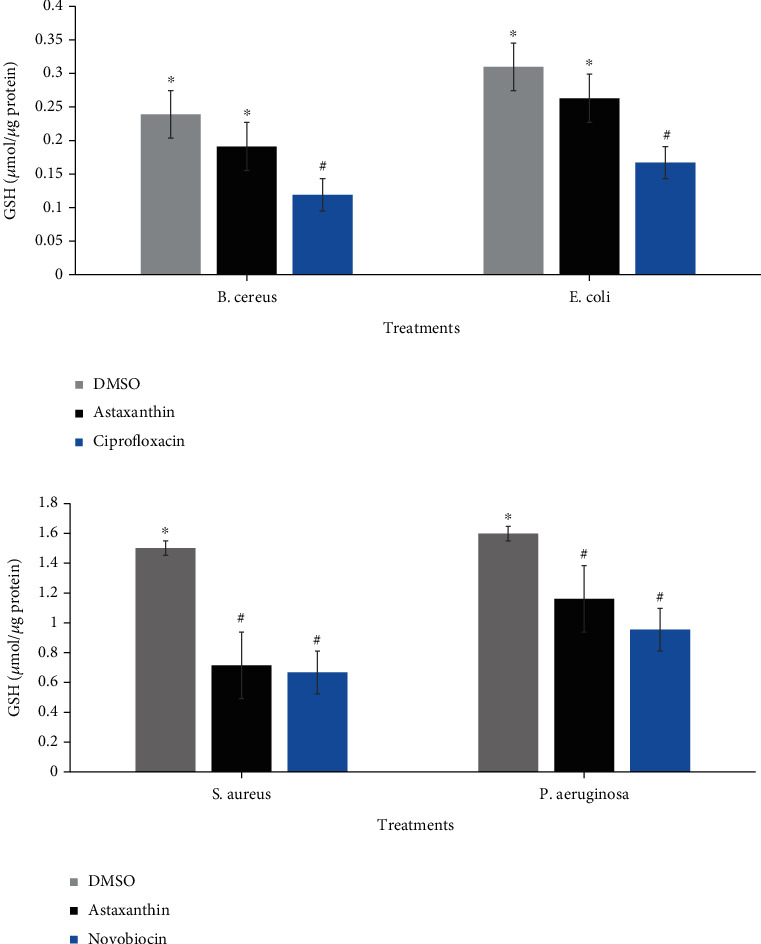
Concentration of reduced glutathione in astaxanthin (4x MIC) treated (a) *B. cereus* and *E. coli* (b) *S. aureus* and *P. aeruginosa*. ^#^^∗^Bars carrying the same symbol are not significantly different (*p* > 0.05).

**Figure 10:**
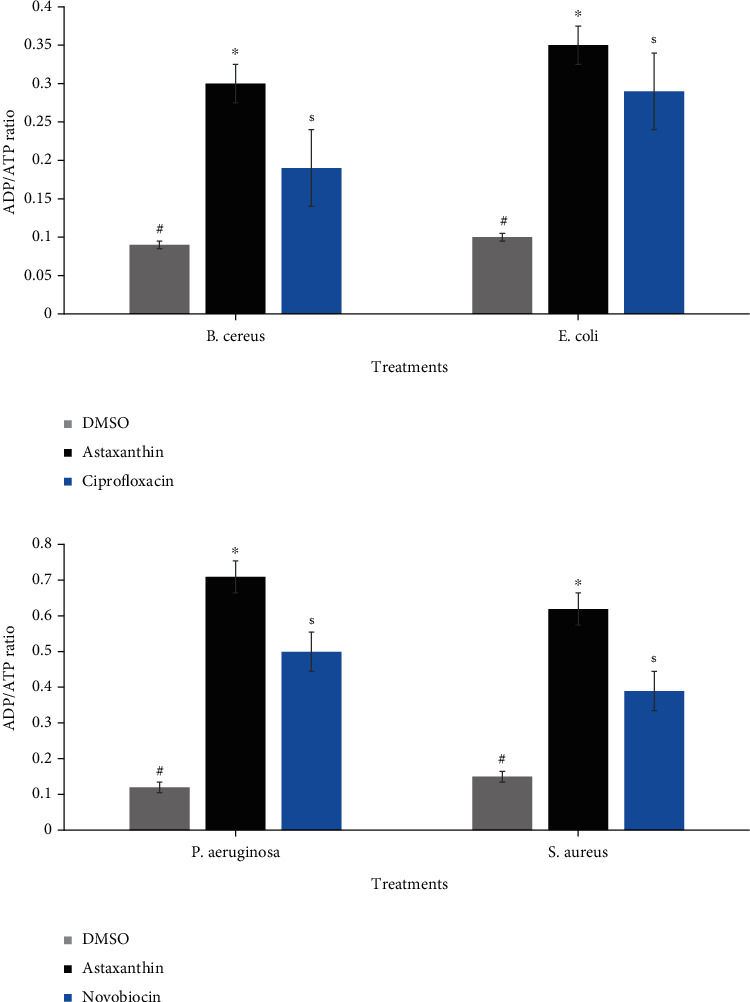
ADP/ATP ratio of (a) *B. cereus* and *E. coli*, exposed to astaxanthin and ciprofloxacin (4x MIC), and (b) *S. aureus and P. aeruginosa* treated with astaxanthin and novobiocin (4x MIC). ^#^^∗^^$^Bars carrying the same symbol are not significantly different (*p* > 0.05).

The data obtained regarding the effect of astaxanthin treatment on GSH concentration of the bacterial cells are presented in Figure 9. Compared with DMSO-treated organisms, the GSH concentration of the astaxanthin-treated cells significantly decreased (*p* ≤ 0.05). However, the decrease in GSH concentration in the reference antibiotics treated cells was more significant than those of the astaxanthin-treated cells except in *S. aureus* where the effect potentiated by astaxanthin compared favorably with that of novobiocin (Figure 9(b)). On the other hand, a significant increase was noted in ADP/ATP ratio following treatment of the bacterial cells with astaxanthin relative to the DMSO-treated cells (Figure 10). It is also noteworthy that the increase in ADP/ATP ratio of astaxanthin-treated cells was higher than those observed with both ciprofloxacin (Figure 10(a)) and novobiocin (Figure 10(b)).

## 4. Discussion

Fluoroquinolones are currently the only bactericidal antibacterials that directly impede bacterial DNA synthesis [27]. They function through generation of “poison” complexes between topo2As and DNA with evidence of ROS/oxidative stress involvement [3, 4, 22, 28]. In this work, the involvement of oxidative stress in antibacterial activity of astaxanthin was investigated *in vitro* and *in silico*.

Molecular docking is an *in silico* method that predicts ligand orientation and conformation in the active site of a receptor, thus allowing for the estimation of binding affinity [29, 30]. In comparison to the reference standards, the higher binding energy observed with astaxanthin against DNA GyrA and topoi IV Par C/ParE in this study could be an indication that astaxanthin had greater binding efficiency and affinity for these proteins than ciprofloxacin and novobiocin. Similarly, judging by the docking scores, both astaxanthin and novobiocin had similar affinity for DNA GyrB. However, since the prediction accuracy of molecular docking is limited due to its simple scoring functions in predicting ligand's interactions in receptors binding pocket [31, 32], the best complex poses in each case were further subjected to molecular dynamic simulations (MDS). The MDS takes into consideration the physical movements of atoms of the ligands and proteins allowing for the estimation of free binding energy, ROG and RMSD [33]. In this study, the higher negative free binding energy score observed with astaxanthin against topoi IV ParC and ParE than the reference antibiotics could be an indication that astaxanthin had better inhibitory effect on these proteins with a higher binding affinity and possibly better stability of the resulting complex than the reference antibiotics. This observation agrees with previous findings where antimicrobial compounds such as withasomnine and garcinol [27, 34] were reported to have potential stronger affinities against topoi IV over synthetic inhibitors. This is however in sharp contrast to the higher binding affinity indicative of better inhibition of DNA GyrA and GyrB with the reference antibiotics than astaxanthin observed in this study. The RMSD measures the thermodynamic conformational stability of protein-ligand complex during MDS and the lower the RMSD the greater the stability [33]. The observation that the average RMSD values of astaxanthin complexes with GyrA, ParC, and ParE were less than the acceptable limit of <3.5 Å and relatively similar to the value obtained with the unbound proteins was a pointer to the fact that astaxanthin does not perturb the conformational stability of these proteins and an indication of better benefit as prospective lead and potential new inhibitor of topoi IV ParC and ParE when considered alongside the higher free binding energy of astaxanthin against these proteins than the values observed with the reference antibiotics. Consistently, the observed higher RMSD of astaxanthin in complex with GyrB when compared with other complexes was suggestive of lower stability of astaxanthin with GyrB, and this was consistent with the lower negative binding free energy obtained for this complex in this study. In sharp contrast to this observation, the lower RMSD value for novobiocin implied that it had a better capacity to improve structural stability of GyrB than astaxanthin. The RoG evaluates the compactness and stability of the resulting complex during MDS, and a more stable complex is usually indicated by a lower RoG value [20]. In this study, the observed relatively similar average RoG values of astaxanthin with unbound protein and reference antibiotics in complexation with GyrA, GyrB, PaC, and ParE further support the RMSD results. Generally, based on the observed findings from the *in silico* evaluations in this study, astaxanthin seems to have more affinity and higher inhibitory potential against the topo2As druggable target of Gram-positive organism (topoi IV ParC/ParE) than the Gram-negative bacteria (DNA GyrA/GyrB).

The free binding energy of a complex is usually attributable to the bond interactions between a receptor and its ligands [35, 36], and the observed higher binding affinity and stability of astaxanthin complex with Topoi IV ParC and Topoi IV ParE in this study could have been due to its higher number of bond interactions with these proteins relative to the interactions formed with the reference antibiotics. This observation is consistent with a previous report where higher binding affinity and stability of five flavonoids against penicillin binding protein 2a were attributed to higher number of established bonds at the binding pockets of the protein [33]. This perhaps could explain why astaxanthin had higher inhibitory potential towards topoi IV ParC and ParE than DNA GyrA and GyrB in this study. Furthermore, the identification of Lys113, Phe115, Ser114, Ala116, Pro 112, and Asp110 residues within the 100-122 loop of topoi IV ParC (Figure S2a) could be another supporting evidence for structural stability of astaxanthin's complex with the protein, as this loop has been reported to be germane in topoi IV ParC stabilization [37]. Although, some of these amino acid residues were also observed in topoi IV ParC complexed with ciprofloxacin, however, within the 100-122 loop, ciprofloxacin had lesser amino acid residues and could have probably contributed to the lower free binding energy observed with ciprofloxacin relative to astaxanthin. Also, Arg65 of topoi IV ParC which formed two hydrogen bonds with astaxanthin was also identified in this study as important amino acid contributing to the stability of the complex. Similarly, for GyrA, the Asp87 (Figure S1b) which formed carbon-hydrogen bond with ciprofloxacin in this study has been shown by Huang [38] to be one of the most important catalytic amino acid residues of GyrA. This important amino acid was absent in the complex with astaxanthin and could have contributed to its lower free binding energy relative to ciprofloxacin observed in this study. For GyrB, amino acid such as Arg20, Pro23, Gln135, and Asp29 (Figure S1c) forming hydrogen bonds and alkyl bonds with astaxanthin was observed to be important residues contributing to the stability of the complex. In contrast to this observation, Gjorgjieva et al. [39] have previously noted Val71, Thr165, Asn46, Pro79, Ile78, Asp73, Glu50, Arg76, Val120, Val43, Val 43, Val164, and Ala 47 to be important residues contributing to the stability of benzothiazole scaffold-based Gyr B inhibitor. Nevertheless, residue Asp73 of GyrB formed van dan Waals interactions with astaxanthin and novobiocin, respectively (Figure S1c and d). Regarding topoi IV ParE, Arg1131, Val1153, and Val1136 that formed hydrogen and Pi-alkyl bonds at the active site with astaxanthin (Figures S2c) were identified as catalytically important residues, though, not in agreement with Gly71, Arg72, Gly73, Ile68, Arg72, Val39, Ile40, Ser43, and Val44 previously reported by Li et al. [40].

The Lipinski's rule of five describes the druggability and oral bioavailability of a biologically active compound [41]. Based on this rule, the drug likeliness as a function of pharmacokinetic traits of a compound or drug candidate can be predicted [42]. In this study, the number of hydrogen bond acceptors and donors for astaxanthin is within the acceptable Lipinski limit of ≤5 and 10, respectively, suggesting a good blood–brain barrier (BBB) permeation effect. The molecular weight of astaxanthin was greater than the Lipinski's value of <500 g/mol but within the recommended range of 130 to 725 g/mol [42] indicating that astaxanthin can still penetrate target cell membrane. Metabolism by the CYP isoenzymes is a key determinant of drug interactions and an indication of drug toxicity [33]. The observation that astaxanthin was predicted to be a noninhibitor of all the CYP isoenzymes in this study is suggestive of its tendency not to cause drug-drug interactions when coadministered with drugs normally metabolized by the enzymes, and this is in tandem with a previous work [43], where related findings were reported in the evaluation of *α*-naphthoflavones against the CYP isoenzymes. Judging by the LD_50_ values and the class of drugs evaluated in this study, astaxanthin stands the chance of being considered suitable for use as a drug candidate relative to the standards that belong to class 4 with higher lethality profile [33].

Since astaxanthin showed some level of significant inhibitory effects especially on the topoi IV ParC and ParE *in silico*, the *in vitro* evaluations were undertaken to confirm the observed effects and the possibility of oxidative stress involvement in its bacterial lethality. Studies have reported the antimicrobial properties of astaxanthin [44–49] with evidence pointing towards its dose-dependent bactericidal and bacteriostatic activity against both Gram-negative and Gram-positive bacteria. Findings from this study corroborate these observations as astaxanthin was found to be potent against both Gram-negative and Gram-positive organisms, and the time-kill susceptibility test of astaxanthin demonstrated a concentration-dependent decrease in bacterial viability. Interestingly too, the lower MIC value of astaxanthin against *S. aureus* than novobiocin in this study suggests that a lower concentration of astaxanthin is required to inactivate the organism and further supports the *in silico* results, where astaxanthin had higher affinity for the topo2As druggable targets (topoi IV ParC/ParE) in Gram-positive organisms than Gram-negative targets (GyrA/GyrB).

Studies have reported an increase in the production of ROS in response to antimicrobials treatment, with the resultant effect being redox homeostasis imbalance [50–54]. This imbalance is caused by increased bacterial respiratory chain activity, which results in oxidative damage to macromolecules (lipids, nucleic acids, and proteins) and consequently cell death [24, 53, 54]. In this study, the observed increase in the level of superoxide anion radicals produced following treatment with astaxanthin could be indicative of ROS generation and its subsequent involvement in bacterial lethality. This was further supported by the decrease in bacterial viability attributable to the inhibitory effect of astaxanthin on hydroxyl radicals in the presence of 2,2′-dipyridyl, a chelator of Fe^2+^ that inhibits Fenton reaction, and consequently hydroxyl radicals' formation. These findings are consistent with the report of Ajiboye et al. [24], where protocatechuic acid enhanced ROS generation, with concomitant damaging effect on the Fe-S cluster proteins of the treated bacterial cells, resulting in either inactivation or death. The ADP/ATP ratio is another plausible marker of oxidative stress, and the cellular respiratory intensity is directly proportional to ADP values [4]. The observed increases in the ADP/ATP ratios in the astaxanthin-treated cells in this study could be an indication of induced oxidative stress and intense cellular respiration in the bacterial cells. This observation is in agreement with the findings of Lobritz et al. [54], where cell death was associated with accelerated respiration, while further lending credence to the contributory role of ROS to antibacterial potential of astaxanthin against the tested strains.

The bacterial systems are equipped with GSH, a nonenzymatic antioxidant responsible for the detoxification of free radicals [55]. During cellular metabolism and in the advent of oxidative stress induction, the antioxidant systems such as GSH of the bacterial cells become depleted in an attempt to detoxify generated ROS. This GSH depletion could promote redox imbalance in bacterial cells in a manner that the cells may not be able to cope with noxious ROS, which will enhance macromolecular cellular damage resulting to death [24, 51]. In this study, the significantly reduced GSH level in the astaxanthin-treated cells is not only indicative of GSH depletion in response to both superoxide and hydroxyl ions produced but supportive evidence that ROS generation was involved in astaxanthin-mediated bacterial lethality. Similar observations regarding GSH have also been reported following treatment of clinically important pathogenic bacteria with plant secondary metabolites [22, 51].

## 5. Conclusion

This study has demonstrated the significance of oxidative stress in astaxanthin-mediated bacterial killing as revealed from the increased ROS generated following treatment with astaxanthin through rate of killing, reduction in GSH, and the corresponding significant increase in ATP/ADP ratio of the bacterial cells. Although astaxanthin had inhibitory effect against both Gram-positive and Gram-negative bacteria, its effects were more pronounced and significant against the Gram-positive organisms *in vitro*, and this observation agrees with the results of the *in silico* analyses regarding the binding free energy, structural stability, and compactness of astaxanthin-topoi IV ParC and ParE complexes, which were higher and better than its effect against GyrA and GyrB. Consequent upon the foregoing and the good pharmacokinetic traits alongside its drug likeliness properties, astaxanthin could be harnessed to develop novel therapeutic candidates against topo2As taking advantage of oxidative stress involvement in its bacterial lethality.

## Figures and Tables

**Table 1:** Docking scores of astaxanthin, ciprofloxacin, and novobiocin against DNA GyrA/GyrB and topo IV ParC/ParE.

Targets	Compound	Docking score (kcal/mol)
DNA gyrase subunit A	Ciprofloxacin	-7.4
	Astaxanthin	-8.8
DNA gyrase subunit B	Novobiocin	-8.7
	Astaxanthin	-8.7
Topoisomerase ParC	Ciprofloxacin	-6.9
	Astaxanthin	-8.4
Topoisomerase IV ParE	Novobiocin	-6.6
	Astaxanthin	-6.7

**Table 2:** Binding free energy scores of astaxanthin, ciprofloxacin, and novobiocin against DNA GyrA/GyrB and topo IV ParC/ParE.

Components of energy (kcal/mol)					
Complex	Δ *E*_vdW_	Δ*E*_elec_	Δ*G*_gas_	Δ*G*_solv_	Δ*G*_bind_
DNA Gyr subunit A					
AST	−91.94 ± 6.335	−48.45 ± 2.509	−29.39 ± 4.447	160.55 ± 2.716	−28.84 ± 4.830
CIP	−51.64 ± 7.683	−179.07 ± 11.782	−184.72 ± 8.341	120.99 ± 7.618	−33.72 ± 5.334
DNA Gyr subunit B					
AST	−36.0 ± 11.341	−16.97 ± 2.760	−52.98 ± 8.410	28.55 ± 9.481	−24.42 ± 3.112
NOV	−62.44 ± 5.754	−85.90 ± 14.312	−148.34 ± 24.400	91.81 ± 13.968	−56.52 ± 11.450
Topoisomerase ParC					
CIP	−14.09 ± 0.289	−73.84 ± 3.193	−87.94 ± 4.301	77.945 ± 12.743	−10.19 ± 4.771
AST	−48.57 ± 5.207	−7.58 ± 0.273	−56.15 ± 8.337	20.60 ± 5.699	−35.55 ± 5.034
Topoisomerase ParE					
NOV	−31.43 ± 3.522	−14.11 ± 3.943	−45.83 ± 8.001	27.19 ± 7.332	−18.63 ± 3.526
AST	−48.87 ± 5.100	−20.50 ± 3.943	−69.38 ± 13.704	30.64 ± 9.644	−38.73 ± 5.191

Δ*E*_vdW_: van der Waals energy; Δ*E*_elec_: electrostatic energy; Δ*E*_gas_: gas phase free energy; Δ*G*_sol_: solvation free energy; Δ*G*_bind_: total binding free energy; AST: astaxanthin; CIP: ciprofloxacin; NOV: novobiocin.

**Table 3:** ADMET properties of astaxanthin and reference antibiotics.

Ligands	MW <500 (g/mol)	HB − A ≤ 10	HB − D ≤ 5	Log *P*_o/w_ ≤ 5	WS	GI absorption	BBB permeant	pgp	Inhibitor of CYP 450 s					LV	BS	H	C	IM	M	CY	LD 50 (mg/kg)	TC
									CYP 1A2	CYP 2C19	CYP 2C9	CYP 2D6	CYP 3A4									
Astaxanthin	596.84	4	2	6.63	MS	Low	N	Y	N	N	N	N	N	2	0.17	I	I	I	I	I	4600	5
Novobiocin	612.62	11	5	3.23	MS	Low	N	N	N	N	N	N	Y	2	0.17	I	I	A	I	I	962	4
Ciprofloxacin	331.34	5	2	2.24	S	High	N	Y	N	N	N	N	N	0	0.55	I	I	I	A	I	2000	4

MW: molecular weight; HB-A: hydrogen bond acceptor; HB-D: hydrogen bond donor; Pgp substrate: permeability glycoprotein substrate; WS: water solubility; CYP: cytochrome; MS: moderately soluble; GI absorption: gastrointestinal absorption; S: soluble; N: no; Y: yes; I: inactive; A: active; LV: Lipinski violations; BS: bioavailability score; H: hepatotoxicity; C: carcinogenicity; IM: immunotoxicity; M: mutagenicity; CY: cytotoxicity; LD: lethal dose; TC: toxicity class; BBB permeant: blood-brain barrier permeation; Log P_o/w_: partition coefficient.

**Table 4:** MIC and MBC of astaxanthin against Gram-positive (*S. aureus* and *B. cereus*) and Gram-negative (P. *aeruginosa* and *E. coli*) strains.

Bacterial strain	Ciprofloxacin (*μ*g/ml)		Novobiocin (*μ*g/ml)		Astaxanthin (*μ*g/ml)	
	MIC	MBC	MIC	MBC	MIC	MBC
*Bacillus cereus*	0.125	0.5	0.25	32	16	32
*Escherichia coli*	0.125	0.5	0.25	32	16	32
*Pseudomonas aeruginosa*	0.125	0.5	0.125	32	16	32
*Staphylococcus aureus*	0.125	0.25	16	64	8	32

## Data Availability

The data used to support the findings of this study are included within the article.

## Supplementary Materials

Supplementary Materials Additional data for the molecular docking are presented in the supplementary file (Supplementary Table S1 and Figures S1 and S2).

## References

[1] Sebbane F., Lemaître N. (2021). Antibiotic therapy of plague: a review. *Biomolecules*.

[2] Ibrahim M. E., Bilal N. E., Hamid M. E. (2013). Increased multi-drug resistant <i>Escherichia coli</i> from hospitals in Khartoum state, Sudan. *African Health Sciences*.

[3] Eghbaliferiz S., Iranshahi M. (2016). Prooxidant activity of polyphenols, flavonoids, anthocyanins and carotenoids updated review of mechanisms and catalyzing metals. *Phytotherapy Research*.

[4] Ajiboye T. O., Skiebe E., Wilharm G. (2018). Phenolic acids potentiate colistin-mediated killing of Acinetobacter baumannii by inducing redox imbalance. *Biomedicine and Pharmacotherapy*.

[5] Dwyer D. J., Kohanski M. A., Hayete B., Collins J. J. (2007). Gyrase inhibitors induce an oxidative damage cellular death pathway inEscherichia coli. *Molecular Systems Biology*.

[6] Alt S., Mitchenall L. A., Maxwell A., Heide L. (2011). Inhibition of DNA gyrase and DNA topoisomerase IV of Staphylococcus aureus and Escherichia coli by aminocoumarin antibiotics. *Journal of Antimicrobial Chemotherapy*.

[7] Pham T. D., Ziora Z. M., Blaskovich M. A. T. (2019). Quinolone antibiotics. *Medicinal Chemistry Communications*.

[8] Ashley R., Dittmore A., McPherson S. A., Turnbough C. L., Neuman K. C., Osheroff N. (2017). Activities of gyrase and topoisomerase IV on positively supercoiled DNA. *Nucleic Acids Research*.

[9] Madabhushi R. (2018). The roles of DNA topoisomerase II*β* in transcription. *International Journal of Molecular Sciences*.

[10] Kohanski M., Dwyer D., Collins J. (2010). How antibiotics kill bacteria: from targets to networks. *Nature Reviews Microbiology*.

[11] Wang X., Zhao X., Malik K., Drlica K. (2010). Contribution of reactive oxygen species to pathways of quinolone-mediated bacterial cell death. *Journal of Antimicrobial Chemotherapy*.

[12] Galati G., Sabzevari O., Wilson J. X., O’Brien P. J. (2002). Prooxidant activity and cellular effects of the phenoxyl radicals of dietary flavonoids and other polyphenolics. *Toxicology*.

[13] Gibellini L., Bianchini E., De Biasi S., Nasi M., Cossarizza A., Pinti M. (2015). Natural compounds modulating mitochondrial functions. *Evidence-Based Complementary and Alternative Medicine*.

[14] Yang L., Mih N., Anand A. (2019). Cellular responses to reactive oxygen species are predicted from molecular mechanisms. *Proceedings of the National Academy of Sciences of the United States of America*.

[15] Davinelli S., Nielsen M. E., Scapagnini G. (2018). Astaxanthin in skin health, repair and disease: a comprehensive review. *Nutrients*.

[16] Ambati R. R., Phang S. M., Ravi S., Aswathanarayana R. G. (2014). Astaxanthin: sources extraction stability, biological activities and its commercial applications - a review. *Marine Drugs*.

[17] Ellis C. R., Kruhlak N. L., Kim M. T., Hawkins E. G., Stavitskaya L. (2018). Predicting opioid receptor binding affinity of pharmacologically unclassified designer substances using molecular docking. *PLoS One*.

[18] Trott O., Olson A. J. (2009). AutoDockVina: improving the speed and accuracy of docking with a new scoring function, efficient optimization, and multithreading. *Journal of Computational Chemistry*.

[19] Sabiu S., Idowu K. (2021). An insight on the nature of biochemical interactions between glycyrrhizin, myricetin and CYP3A4 isoform. *Journal of Food Biochemistry*.

[20] Sabiu S., Balogun F. O., Amoo S. O. (2021). Phenolics Profiling of Carpobrotus edulis (L.) N.E.Br. and Insights into Molecular Dynamics of Their Significance in Type 2 Diabetes Therapy and Its Retinopathy Complication. *Molecules*.

[21] Teh C. H., Nazni W. A., Nurulhusna A. H., Norazah A., Lee H. L. (2017). Determination of antibacterial activity and minimum inhibitory concentration of larval extract of fly via resazurin-based turbidometric assay. *BMC Microbiology*.

[22] Oloyede H. O. B., Ajiboye H. O., Salawu M. O., Ajiboye T. O. (2017). Influence of oxidative stress on the antibacterial activity of betulin, betulinic acid and ursolic acid. *Microbial Pathogenesis*.

[23] Ajiboye T. O., Naibi A. M., Abdulazeez I. O. (2016). Involvement of oxidative stress in bactericidal activity of 2-(2-nitrovinyl) furan against *Escherichia coli* , *Pseudomonas aeruginosa* and *Staphylococcus aureus*. *Microbial Pathogenesis*.

[24] Ajiboye T. O., Habibu R. S., Saidu K. (2017). Involvement of oxidative stress in protocatechuic acid-mediated bacterial lethality,. *Microbiology Open*.

[25] Tipple T. E., Rogers L. K. (2012). Methods for the determination of plasma or tissue glutathione levels. *Methods in Molecular Biology*.

[26] Mempin R., Tran H., Chen C., Gong H., Kim Ho K., Lu S. (2013). Release of extracellular ATP by bacteria during growth. *BMC Microbiology*.

[27] di Micco S., Masullo M., Bandak A. F. (2019). Garcinol and related polyisoprenylated benzophenones as topoisomerase II inhibitors: biochemical and molecular modeling studies. *Journal of Natural Products*.

[28] Dwyer D. J., Kohanski M. A., Collins J. J. (2009). Role of reactive oxygen species in antibiotic action and resistance. *Current Opinion in Microbiology*.

[29] Kitchen D. B., Decornez H., Furr J. R., Bajorath J. (2004). Docking and scoring in virtual screening for drug discovery: methods and applications. *Nature Reviews Drug Discovery*.

[30] Young D. C. (2009). *Computational drug design: a guide for computational and medicinal chemists*.

[31] Ramírez D., Caballero J. (2016). Is it reliable to use common molecular docking methods for comparing the binding affinities of enantiomer pairs for their protein target. *International Journal of Molecular Sciences*.

[32] Nasution M. A. F., Toepak E. P., Alkaff A. H., Tambunan U. S. F. (2018). Flexible docking-based molecular dynamics simulation of natural product compounds and Ebola virus Nucleocapsid (EBOV NP): a computational approach to discover new drug for combating Ebola. *BMC Bioinformatics*.

[33] Verma A. K., Ahmed S. F., Hossain M. S. (2021). Molecular docking and simulation studies of flavonoid compounds against PBP-2a of methicillin‐resistantStaphylococcusaureus. *Journal of Biomolecular Structure and Dynamics*.

[34] George T. K., Tomy A., Jisha M. S. (2020). Molecular docking study of bioactive compounds of Withania somnifera extract against topoisomerase IV Type B. *Proceedings of the National Academy of Sciences, India Section B: Biological Sciences*.

[35] Izadi H., Stewart K. M. E., Penlidis A. (2014). Role of contact electrification and electrostatic interactions in gecko adhesion. *Journal of the Royal Society Interface*.

[36] Park S. J., Seo M. K. (2011). Solid-solid interfaces. *Interface Science and Composites*.

[37] Laponogov I., Veselkov D. A., Sohi M. K. (2007). Breakage-reunion domain of Streptococcus pneumoniae topoisomerase IV: crystal structure of a gram-positive quinolone target. *PLoS One*.

[38] Huang W. M. (1994). Type II DNA topoisomerase genes. *Advances in Pharmacology*.

[39] Gjorgjieva M., Tomašič T., Barančokova M. (2016). Discovery of benzothiazole scaffold-based DNA gyrase B inhibitors. *Journal of Medicinal Chemistry*.

[40] Li Y., Wong Y. L., Ng F. M. (2016). *Escherichia coli* Topoisomerase IV E Subunit and an Inhibitor Binding Mode Revealed by NMR Spectroscopy∗. *Journal of Biological Chemistry*.

[41] Lipinski C. A., Lombardo F., Dominy B. W., Feeney P. J. (2001). Experimental and computational approaches to estimate solubility and permeability in drug discovery and development settings^1^. *Advanced Drug Delivery Reviews*.

[42] Dewanjee S., Paul P., Dua T. K., Bhowmick S., Saha A. (2020). Big Leaf Mahogany Seeds. *Nuts and Seeds in Health and Disease Prevention*.

[43] Cui J., Meng Q., Zhang X., Cui Q., Zhou W., Li S. (2015). Design and synthesis of new *α*-naphthoflavones as cytochrome P450 (CYP) 1B1 inhibitors to overcome docetaxel-resistance associated with CYP1B1 overexpression. *Journal of Medicinal Chemistry*.

[44] Veeruraj A., Liu L., Zheng J., Wu J., Arumugam M. (2019). Evaluation of astaxanthin incorporated collagen film developed from the outer skin waste of squid _Doryteuthis singhalensis_ for wound healing and tissue regenerative applications. *Materials Science and Engineering*.

[45] Mosaad Y. O., Gobba N. E., Hussein M. A. (2016). Astaxanthin: a promising protector against gentamicin induced nephrotoxicity in rats. *Current Pharmaceutical Biotechnology*.

[46] Shanmugapriya K., Kim H., Saravana P. S., Chun B., Kang H. W. (2018). Astaxanthin-alpha tocopherol nanoemulsion formulation by emulsification methods: Investigation on anticancer, wound healing, and antibacterial effects. *Colloids and Surfaces B: Biointerfaces*.

[47] Sharma P., Jha A. B., Dubey R. S., Pessarakli M. (2012). Reactive Oxygen Species, Oxidative Damage, and Antioxidative Defense Mechanism in Plants under Stressful Conditions. *Journal of Botany*.

[48] Weintraub S., Shpigel T., Harris L. G., Schuster R., Lewis E. C., Lewitus D. Y. (2017). Astaxanthin-based polymers as new antimicrobial compounds. *Polymer Chemistry*.

[49] Ushakumari U. N., Ramanujan R. (2013). Isolation of astaxanthin from marine yeast and study of its pharmacological activity. *International Current Pharmaceutical Journal*.

[50] Ajiboye T. O., Haliru F. Z. (2016). Redox and respiratory chain related alterations in the lophirones B and C-mediated bacterial lethality. *Microbial Pathogenesis*.

[51] Belenky P., Ye J. D., Porter C. B. (2015). Bactericidal antibiotics induce toxic metabolic perturbations that lead to cellular damage. *Cell Reports*.

[52] Dwyer D. J., Belenky P. A., Yang J. H. (2014). Antibiotics induce redox-related physiological alterations as part of their lethality. *Proceedings of the National Academy of Sciences of the United States of America*.

[53] Kohanski M. A., Dwyer D. J., Hayete B., Lawrence C. A., Collins J. J. (2007). A common mechanism of cellular death induced by bactericidal antibiotics. *Cell*.

[54] Lobritz M. A., Belenky P., Porter C. B. M. (2015). Antibiotic efficacy is linked to bacterial cellular respiration. *Proceedings of the National Academy of Sciences*.

[55] Sannasimuthu A., Sharma D., Paray B. A., Al-Sadoon M. K., Arockiaraj J. (2020). Intracellular oxidative damage due to antibiotics on gut bacteria reduced by glutathione oxidoreductase-derived antioxidant molecule GM15. *Archives of Microbiology*.

