# Supplementary material for: Astaxanthin-Mediated Bacterial Lethality: Evidence from Oxidative Stress Contribution and Molecular Dynamics Simulation
Source: Oxid Med Cell Longev. 2021 Dec 9;2021:7159652. doi: 10.1155/2021/7159652 (PMC8677388; doi:10.1155/2021/7159652)
Supplement: Supplementary Materials — Additional data for the molecular docking are presented in the supplementary file (Supplementary Table S1 and Figures S1 and S2). [file 7159652.f1.docx]

Table S1. Grid box values ensuring coverage of the ligand binding sites in the 3Ds protein structure for docking.

| **COMPLEXES** | **CENTER** | | | **SIZE** | | |
| --- | --- | --- | --- | --- | --- | --- |
|  | **X** | **Y** | **Z** | **X** | **Y** | **Z** |
| GyrA + Ciprofloxacin | 159.179 | 160.214 | 195.814 | 60.471 | 87.4973 | 127.529 |
| GyrA + Astaxanthin | 151.441 | 166.285 | 187.404 | 76.3438 | 94.1558 | 148.247 |
| GyrB + Novobiocin | 159.93 | 163.474 | 191.656 | 67.3159 | 54.9684 | 129.352 |
| GyrB + Astaxanthin | 164.541 | 169.504 | 187.524 | 50.3129 | 67.4652 | 121.103 |
| ParC + Ciprofloxacin | -18.2367 | -12.7409 | -3.0182 | 43.9564 | 44.4694 | 36.295 |
| ParC + Astaxanthin | -23.7691 | -10.3856 | -4.95099 | 58.3055 | 52.299 | 37.2601 |
| ParE + Novobiocin | 23.3962 | 30.3307 | 44.9868 | 15.1979 | 28.1027 | 35.6674 |
| ParE + Astaxanthin | 21.1119 | 27.2523 | 45.8463 | 39.8304 | 48.2487 | 37.9457 |


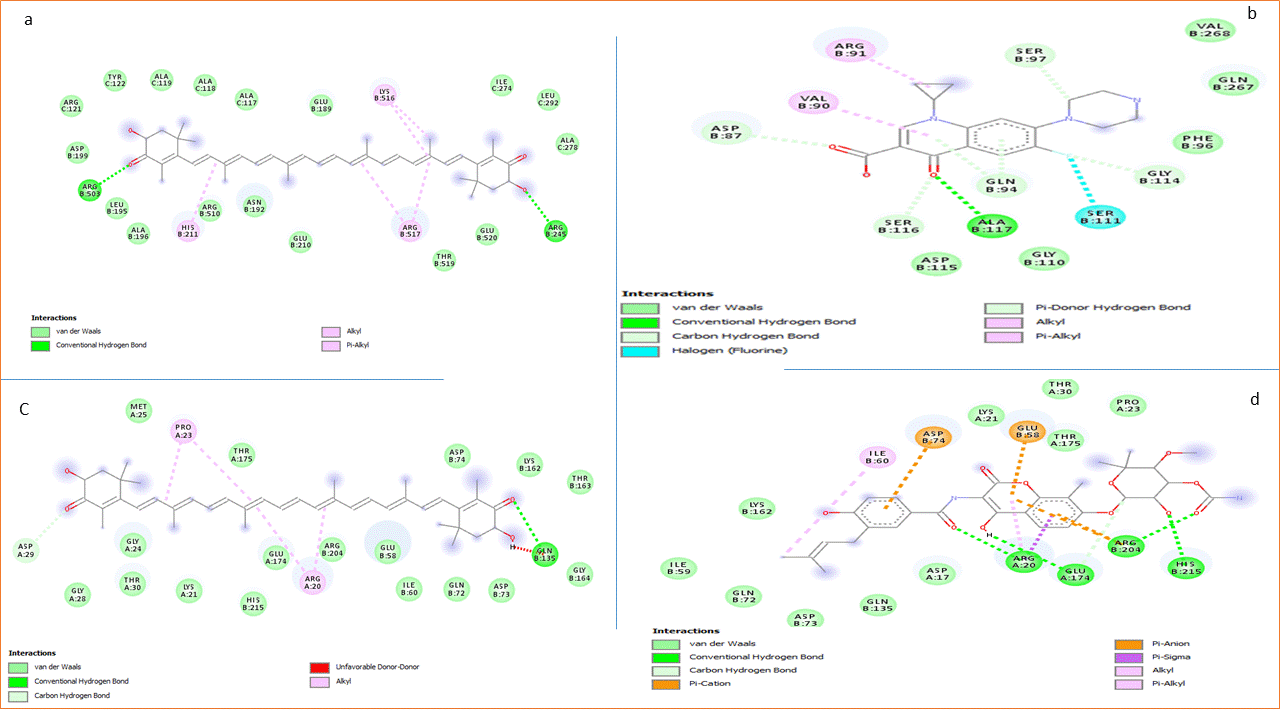


Figure S1: Interaction plots of (a) Docked DNA gyrase A + Astaxanthin, (b) Docked DNA gyrase A + Ciprofloxacin, (c) Docked DNA gyrase B + Astaxanthin, and (d) Docked DNA gyrase B + Novobiocin,


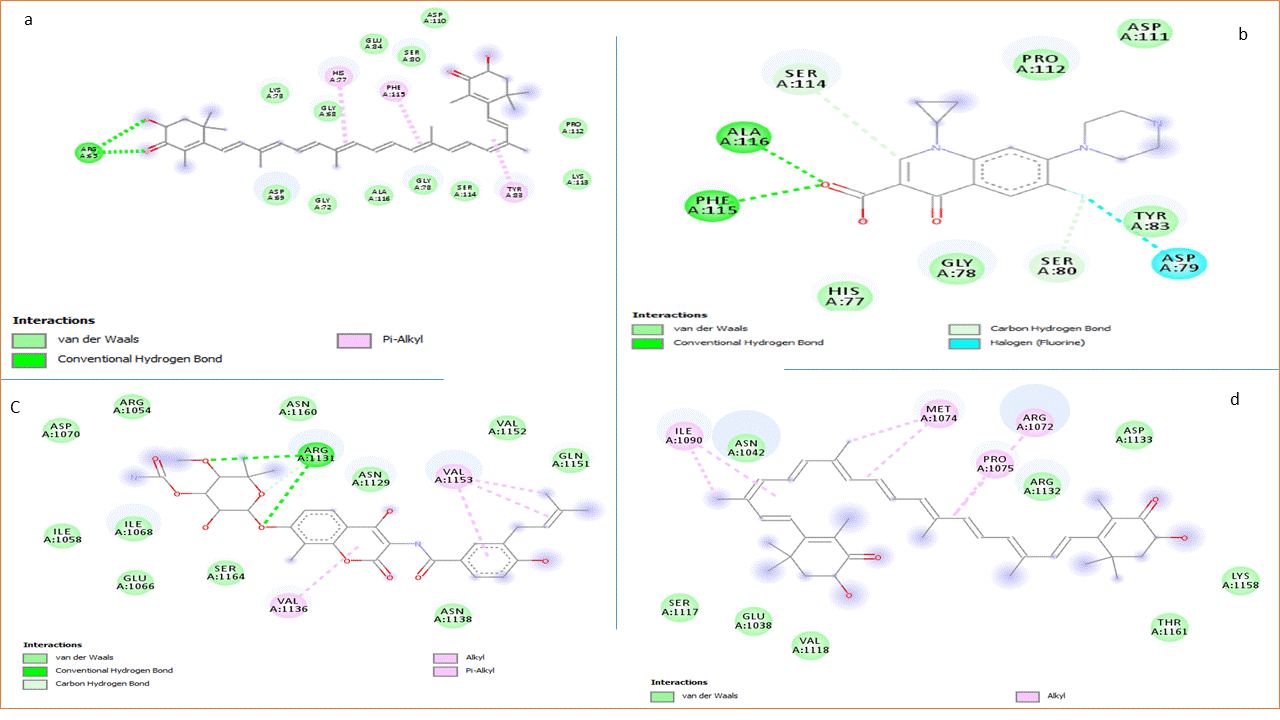


Figure S2: Interaction plots of (a) Docked Topoi IV ParC + Astaxanthin, (b) Docked Topoi IV ParC + Ciprofloxacin, (c) Docked Topoi IV ParE + Astaxanthin, and (d) Docked Topoi IV ParE + Novobiocin
